# An Appraisal of the Influence of the Metabotropic Glutamate 5 (mGlu5) Receptor on Sociability and Anxiety

**DOI:** 10.3389/fnmol.2019.00030

**Published:** 2019-02-19

**Authors:** Arnau Ramos-Prats, Julia Kölldorfer, Elena Paolo, Maximilian Zeidler, Gabriele Schmid, Francesco Ferraguti

**Affiliations:** Department of Pharmacology, Medical University of Innsbruck, Innsbruck, Austria

**Keywords:** social behavior, glutamate receptors, anxiety, brain networks, MTEP

## Abstract

Amongst the many neurotransmitter systems causally linked to the expression of social behavior, glutamate appears to play a pivotal role. In particular, metabotropic glutamate 5 (mGlu5) receptors have received much attention as its altered function has been reported in several mouse models of autism spectrum disorders and mental retardation. Inhibition of the activity of mGlu5 receptors by means of genetic or pharmacological manipulations improved social deficits in some of these animal models. However, in normal wild-type (WT) mice, pharmacological blockade of mGlu5 receptors yielded inconsistent results. The aim of our study was to investigate the actual contribution of decreased or absent mGlu5 receptor function in sociability and anxiety-like behavior as well as to explore the impact of mGlu5 receptor ablation on the pattern of brain activation upon social exposure. Here we show that *Grm5*-/- mice display higher social preference indexes compared to age-matched WT mice in the three-chambered social task. However, this effect was accompanied by a decreased exploratory activity during the test and increased anxiety-like behavior. Contrary to mGlu5 receptor ablation, the mGlu5 receptor negative allosteric modulator 3-((2-methyl-1,4-thiazolyl)ethynyl)pyridine (MTEP) induced anxiolytic effects without affecting social preference in WT mice. By mapping c-Fos expression in 21 different brain regions known to be involved in social interaction, we detected a specific activation of the prefrontal cortex and dorsolateral septum in *Grm5*-/- mice following social interaction. C-Fos expression correlation-based network and graph theoretical analyses further suggested dysfunctional connectivity and disruption of the functional brain network generated during social interaction in *Grm5*-/- mice. The lack of mGlu5 receptors resulted in profound rearrangements of the functional impact of prefrontal and hippocampal regions in the social interaction network. In conclusion, this work reveals a complex contribution of mGlu5 receptors in sociability and anxiety and points to the importance of these receptors in regulating brain functional connectivity during social interaction.

## Introduction

The group I metabotropic glutamate 5 (mGlu5) receptor couples to Gαq/11 proteins to activate a number of intracellular signaling cascades (Hermans and Challiss, 2001; Nicoletti et al., 2011) and regulates synaptic activity and plasticity (Manahan-Vaughan and Braunewell, 2005; Homayoun and Moghaddam, 2010). This receptor is abundantly expressed in telencephalic brain areas (Ferraguti and Shigemoto, 2006) involved in learning and memory, emotions and in the control of movement and it was found to contribute to a variety of behaviors ranging from cognition to sensory-motor gating and novelty-induced locomotion (Lu et al., 1997; Kinney et al., 2003; Brody et al., 2004; Jew et al., 2013).

A large body of evidence has implicated an altered mGlu5 receptor signaling or expression in the pathology of several neuropsychiatric disorders, including autism, schizophrenia, and anxiety (Nicoletti et al., 2011; Bhakar et al., 2012; D’Antoni et al., 2014; Matosin et al., 2017; Ferraguti, 2018). In particular, enhanced mGlu5 receptor activity has been suggested as one of the underlying mechanisms contributing to several symptoms of fragile X (FX) syndrome, the most common inherited form of intellectual disability (Bhakar et al., 2012). FX-like phenotypes, such as impaired sociability, could be corrected in Fmr1 knock-out mice by reducing the activity of mGlu5 receptors using both genetic and chronic pharmacological treatments (Dölen et al., 2007; Thomas et al., 2011; Bhakar et al., 2012; Gantois et al., 2013). Likewise, altered mGlu5 receptor function was reported in other mouse models of autism spectrum disorders (ASD) and mental retardation (Burket et al., 2011; Chung et al., 2015; Tian et al., 2015; Tao et al., 2016; Vicidomini et al., 2017). The “mGlu5 receptor theory of FX” was recently tested in several phase II, placebo controlled, clinical trials, which, however, did not achieve significant efficacy in the primary end point of improvement on behavioral symptoms (Berry-Kravis et al., 2016; Youssef et al., 2018). Despite the big disappointment and conjectures on the validity of the theory, a number of caveats characterizing these trials may explain why they have failed. First and foremost, the scales of treatment response used in these studies could be biased by caregiver or family involvement in ratings, thus lacking truly quantitative and objective measures of behavioral and cognitive performance. These assessment scales are also known to be subject to a strong placebo effect. Moreover, the study duration and the doses of mGlu5 receptor antagonists utilized might have been inadequate based on preclinical animal data. These results, besides highlighting the difficulty of translating findings from animal models to humans, also call for a new appraisal of the role of mGlu5 receptors in distinct behaviors.

For instance, the influence of mGlu5 receptor antagonism on different aspects of social behavior in wild-type (WT) rodents has been poorly investigated and the current literature contains a number of inconsistent results. The negative allosteric modulator (NAM) 3-((2-methyl-1,4-thiazolyl)ethynyl)pyridine (MTEP) was shown to induce social isolation in rats (Koros et al., 2007), whereas both AFQ056/Mavoglurant and 2-methyl-6-(phenylethynyl)pyridine (MPEP), when administered systemically, elicited no substantial effects on sociability in WT mice (Gantois et al., 2013; Chung et al., 2015). It remains, therefore, unclear whether the complex effects of mGlu5 receptor antagonism in social behavior are due to the limited specificity of systemic pharmacological approaches, species differences in receptor occupancy or pharmacokinetic differences of distinct mGlu5 receptor antagonists (Anderson et al., 2003).

Another complex aspect in the study of mGlu5 receptor in social behavior is its controversial interaction with anxiety. Besides ASD, social function is severely affected in patients with anxiety disorders. The high co-morbidity between social deficits and anxiety can be partially explained by the shared circuitry underlying both anxiety-related and social behavior (for review see: Allsop et al., 2014). Several studies have reported anxiolytic properties of different mGlu5 receptor antagonists, both in animal research and in humans (for review see: Ferraguti, 2018). Nonetheless, to which extent mGlu5 receptor modulation simultaneously regulate anxiety and social behavior in healthy and pathological conditions has not been investigated in depth. Only one study has addressed the role of mGlu5 receptors in the acquisition, expression and extinction of social anxiety in rodent models (Slattery et al., 2017). Overall, the role of mGlu5 receptors in social behavior remains unclear. In the present study, we sought to reassess the effect of mGlu5 receptor ablation or mGlu5 receptor negative allosteric modulation in social preference and anxiety-like behavior, using the classical three-chambered social task (Moy et al., 2004; Nadler et al., 2004) and the light-dark test, respectively. Furthermore, we have investigated the influence of mGlu5 receptors on brain activity patterns upon social and non-social investigation using c-Fos expression as a marker of neuronal activation. Based on these data, we further explored interregional functional connectivity using network analysis to understand at the anatomical level where mGlu5 receptors may regulate functional brain connectivity during social exploration.

## Materials and Methods

All procedures involving animals were approved by the Austrian Animal Experimentation Ethics Board and were performed in compliance with the European Convention for the Protection of Vertebrate Animals used for Experimental and Other Scientific Purposes (ETS no. 123). Every effort was taken to minimize the number of animals used. mGlu5 receptor knock-out (*Grm5*-/-) mice (Lu et al., 1997), were backcrossed to C57BL/6J from Charles River Laboratories (Sulzfeld, Germany) for at least 10 generations. Because *Grm5*-/- female mice have deficits in maternal care, breeding was carried out using homozygous male *Grm5*-/- and heterozygous females. Animals were weaned at 4 weeks of age and grouped housed in a climate-controlled facility on a 12 h/12 h light/dark cycle with lights on at 07:00 AM, with water and food *ad libitum*. Genotyping was performed from ear punches and determined by PCR. Since the breeding strategy or postnatal mothering of Grm5-/+ mice were shown not to influence the offspring behavioral phenotype (Brody and Geyer, 2004), age-matched wild type (WT) C57BL/6J mice (purchased from Charles River Laboratories, Sulzfeld) were selected, instead of WT littermates, as control animals for Grm5-/- mice as well as for pharmacological experiments. C57BL/6J mice were allowed to acclimatize for at least 2 weeks before any experimental procedure. Only adult (11–18 weeks old) male mice were used. All experiments were performed during the light cycle. Prior to all experiments, animals were acclimatized to the testing room for at least 2 days.

For pharmacological experiments, mice were injected i.p. with MTEP (Hello Bio; Bristol, United Kingdom; 10 mg/kg diluted in 4% dimethylsulfoxide in saline) or vehicle. Mice were tested 5 min or 1-h post i.p. injection of MTEP. Dose and time post i.p. injection were chosen based on previous studies on behavioral activity and receptor occupancy of MTEP (Anderson et al., 2003; Busse et al., 2004; Nagel et al., 2015).

### Three-Chambered Social Task

Social behavior was assessed by means of a modified three-chambered social task apparatus (Moy et al., 2004; Nadler et al., 2004). The chamber was an opaque glass rectangular box (75 cm long × 30 cm wide × 35 cm tall) divided into three equal compartments, connected through small rectangular doors (7 cm × 7 cm) allowing free access into each chamber. Different illumination conditions were used for pharmacological (infrared light; Lux < 5) and non-pharmacological experiments (Lux < 30). The procedure involved two phases: habituation and sociability. The test mouse was first placed in the middle chamber and allowed to explore all three chambers for 10 min. After this habituation period, a novel unfamiliar mouse (sex, strain and age-matched) was placed into a mesh cylinder (15 cm tall, 7 cm diameter) in the least explored side chamber, whereas an identical empty mesh cylinder was placed in the opposite chamber. The mesh cylinder allowed for air exchange, visual, olfactory and auditory interaction, but prevented fighting. The test mouse was then allowed to explore the chambers for 10 min (sociability). Measurements during the test phase included: distance traveled, latency to explore side chambers at the beginning of the test, time spent in each chamber and in close proximity to the mesh cylinders (<5 cm). Tracking and scoring was performed using Ethovision XT 10 software (Noldus; RRID:SCR_000441). The social preference index for conspecific chamber time was calculated as follows:

$$\begin{array}{c} \frac{\left(Tc-To\right)}{\left(Tc+To\right)}\;\times 100 \end{array}$$

Tc = Time spent in conspecific chamber

To = Time spent in object chamber

whereas the social preference index for time in close interaction was calculated as follows:

$$\begin{array}{c} \frac{\left(Tnc-Tno\right)}{\left(Tnc+Tno\right)}\;\times 100 \end{array}$$

Tnc = Time spent in close proximity to the conspecific

Tno = Time spent in close proximity to the object

### Object Interaction Task

Object interaction was assessed in an identical apparatus as described above. Under infrared light conditions, mice were first allowed to explore the three chambers of the empty apparatus for 10 min. After this habituation, an empty mesh cylinder was placed in the most preferred side chamber and mice were allowed to freely explore the apparatus for 30 min. Tracking and scoring was performed using Ethovision XT 10 software (Noldus; RRID:SCR_000441). Time spent in the object chamber was scored and expressed as the percentage of total test time.

### Light-Dark Test

Anxiety-like behavior was tested using the light-dark test. The apparatus (TSE Systems, Bad Homburg, Germany) consisted of a dark (<10 Lux) “safe” compartment and an illuminated aversive compartment (400 Lux). The compartments were connected by a small opening (7 cm × 7 cm wide) located in the center of the partition at floor level. Animals were individually placed in the apparatus facing the opening to the dark compartment and allowed to freely explore the apparatus for 10 min. Time spent in the light compartment was measured using Ethovision (Noldus). In pharmacological experiments, mice tested 5 min after injection in the three-chambered social task were re-used after 48 h in the light-dark test using a counterbalanced design. Conversely, independent mouse cohorts were used for the 1 h post-injection experiments.

### Forced Social and Object Interaction for c-Fos Induction

Adult male WT and *Grm5*-/- mice were single-housed for 72 h prior to the test. Mice from each genotype were divided into three groups (*n* = 7/group): A group exposed to an empty mesh cylinder (object) in the home cage for 10 *min*, a second group exposed in the home cage to a mesh cylinder enclosing an unfamiliar sex and age-matched mouse (conspecific) for 10 min and a control group maintained undisturbed in the home cage (HC). After the test, the cylinder was removed and the mice left in their home cages undisturbed. Mice were then perfused 2 h after the end of the experimental manipulation. Tracking and scoring of the time spent in close proximity (<3 cm) with the object or conspecific was performed using the Ethovision XT 10 software (Noldus). Measurement of exploration time of the novel object or novel conspecific was obtained from an independent batch of mice from those used for c-Fos quantification.

### Immunocytochemistry

Mice were deeply anesthetized by i.p. injection of Thiopental (150 mg/kg) and were perfused with a fixative made of 4% w/v paraformaldehyde and 15% v/v of a saturated solution of picric acid in phosphate buffer (PB) 0.1 M pH 7.4, for 12 min. Brains were removed from the skull, washed in 0.1 M PB and sliced coronally in 50 μm-thick sections on a vibratome (VT1000S, Leica Microsystems, Vienna, Austria).

Immunocytochemistry was performed as previously described (Sreepathi and Ferraguti, 2012). Briefly, free-floating sections were first washed with Tris-buffered saline (TBS; 0.9% NaCl, pH 7.4) and then incubated in 20% normal horse serum in TBS and 0.3% Triton X100 (TX) for 1 h at room temperature (RT, 21–23°C). After blocking, sections were incubated with a polyclonal goat primary antibody against c-Fos (1:300; Santa Cruz Biotechnology, Santa Cruz, CA, United States, catalog #sc-52, lot #F1112) for ∼72 h at 6°C. After three washes in TBS, the biotinylated secondary antibody (horse anti-goat IgG 1:500, Vector Laboratories, Burlingame, CA, United States, catalog #BA-9500,) was applied overnight at 6°C at a dilution of 1:500 in a buffer with the same composition as for the primary antibody. The sections were then washed and incubated in Vectastain elite ABC complex (diluted 1:100; Vector Laboratories) in TBS at RT for 1 h. Subsequently, the sections were washed in TB several times, pre-incubated with 3,3′-diaminobenzidine (DAB; 0.5 mg/ml) for 10 min and then H_2_O_2_ was added to the solution at a final dilution of 0.003% for 2–5 min. Sections were then washed with TBS, mounted in gelatin onto glass slides, air-dried, and then treated with graded ethanol (50, 70, 90, 95, and 100%) and butyl acetate. Finally, slides were coverslipped with Eukitt (Agar Scientific Ltd., Stansted, United Kingdom).

The following brain structures, relevant for social behavior (Kim et al., 2015), were selected for c-Fos quantification and identified based on the mouse brain atlas of Franklin and Paxinos (2007): medial orbital cortex (MO; bregma between +2,8 and +2,22 mm), prelimbic cortex (PrL; bregma between +2,34 and +1,54 mm), infralimbic cortex (IL; bregma between +1,94 and +1,54 mm), accumbens nucleus shell (AcbSh; bregma between +1,42 and +1,18 mm), accumbens nucleus core (AcbC; bregma between +1,42 and +1,18 mm), lateral septal nucleus intermedial part (LSI; bregma between +0,62 and +0,14 mm), lateral septal nucleus dorsal part (LSD; bregma between +0,62 and +0,14 mm), piriform cortex (Pir; bregma between +0,98 and +0,50 mm), medial septal nucleus (MS; bregma between +0,98 and +0,50 mm), medial preoptic nucleus medial part (MPOM; bregma between +0,0,02 and -0,22 mm), paraventricular thalamic nucleus (PV; bregma between -0,22 and -0,58 mm), paratenial thalamic nucleus (PT; bregma between -0,22 and -0,58 mm), reuniens thalamic nucleus (RE; bregma between -0,46 and -0,70 mm), basolateral amygdala (BLA; bregma between -0,94 and -1,46 mm), dorsal hippocampus (CA1, CA2, CA3, DG, bregma between -1,58 and -1,94 mm), lateral hypothalamic area (LH; bregma between -2,18 and -2,46 mm), posteromedial cortical amygdaloid area (PMCo; bregma between -2,18 and -2,46 mm) and periaqueductal gray (PAG; bregma between -2,92 and -3,16 mm). The number of c-Fos positive cells/area was semi-automatically counted with the Neurolucida software (Version 11, MBF Bioscience, RRID:SCR_001775) coupled to an Olympus BX51 Microscope by an experimenter blinded to the treatment condition and genotype. Each brain area was analyzed bilaterally across at least three sections using a sampling window (200 μm × 200 μm) placed always in the same position within the selected area.

### Brain Network Construction and Graph Theoretical Analysis

Network analyses were performed as previously described (Tanimizu et al., 2017a). Briefly, Pearson *r*-values from interregional c-Fos expression data from home cage, object-exposed and conspecific-exposed groups from both genotypes were obtained and used to generate correlation matrices. In order to compare average correlations between groups/genotypes, *r*-values were transformed to Fischer *Z*-values, statistics were calculated, and values were retransformed to r values for graph representation. To characterize the generated social and object interaction networks in both genotypes, positive (*r* > 0.60) interregional c-Fos correlations with a significance level of *p* < 0.05 were used to generate unweighted network graphs. Community clustering to generate weighted network graphs was performed based on modularity optimization, according to Newman and Girvan (2004). Finally, participation coefficient and within-community *z* scores were calculated as defined in Guimerà and Amaral, 2005 and plotted as described by Tanimizu et al. (2017a) in order to visualize the main hubs in the generated networks. Interregional correlation matrices were obtained with Prism 7 software (GraphPad Software Inc., RRID:SCR_002798). Network construction and visualization were performed in R (version 3.3.3) using the igraph (version 1.1.2; Csardi and Nepusz, 2006) and brainGraph (version 1.0.0) packages.

### Data Analysis

Data were analyzed with the Prism 7 software (GraphPad Software Inc.) using two-tailed Student’s *t*-tests or analysis of variance (one-way or two-way ANOVA, factorial or repeated measures). Whenever an ANOVA resulted significant, Holm–Sidak *post hoc* comparisons were applied to analyze the effects of group, genotype, treatment, time and chamber in the behavioral experiments. Two-way ANOVA followed by a *post hoc* Newman–Keuls comparison was used to analyze the effects of genotype and groups in the c-Fos mapping experiment. Two-way ANOVA followed by a *post hoc* Bonferroni comparison was used to analyze differences in network density. Data were considered significant when *p* < 0.05.

## Results

### Effects of mGlu5 Receptor Ablation on Sociability and Anxiety-Like Behavior

**FIGURE 1:**
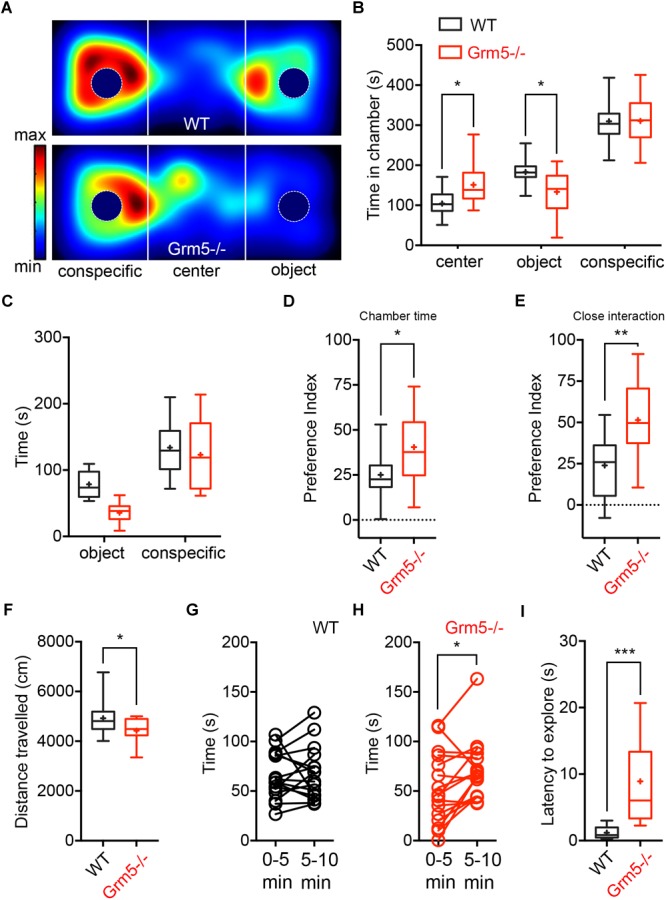
Effects of mGlu5 receptor ablation on sociability. **(A)** Representative heat maps showing time spent by WT mice (upper panel) and *Grm5*–/– (lower panel) mice at each location of the three-chambered apparatus during the test. **(B)** Time spent (s) in the different compartments of the three-chambered apparatus by *Grm5*–/– (*n* = 16) and WT mice (*n* = 16) during the test. **(C)** Time spent (s) in close proximity to the social (conspecific) and non-social (object) stimulus. **(D)** Preference index derived from the numerical difference between time spent in conspecific and object chamber divided by total time spent × 100; and **(E)** index derived from the numerical difference between time spent in close proximity to conspecific and object divided by total time spent in close proximity × 100. **(F)** Distance traveled during the test by *Grm5*–/– and WT mice. **(G)** Time spent in close proximity to the conspecific during the first and last 5 min of the test by WT and **(H)** *Grm5*–/– mice. **(I)** Latency (s) to explore side chambers at the beginning of the three-chambered social task was longer for *Grm5*–/– mice. Boxplots represent median, upper and lower quartiles with 10th and 90th percentile whiskers. Mean is represented with a cross. ^∗^*p* < 0.05, ^∗∗^*p* < 0.01, ^∗∗∗^*p* < 0.001.

**FIGURE 2:**
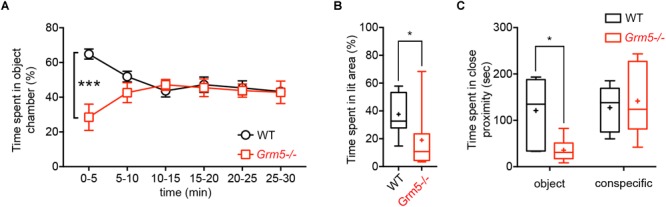
Effects of mGlu5 receptor ablation on non-social exploration and anxiety-like behavior. **(A)** Time spent in object chamber (%) by WT and *Grm5*–/– mice during a 30 min object interaction test in the three-chambered apparatus (*n* = 9/genotype). Points represent mean ± SEM. **(B)** Time spent in the lit area (%) by WT and *Grm5*–/– mice in a Light-dark Test (*n* = 12/genotype). **(C)** Time spent in close proximity to an object (empty mesh cylinder) or to an encaged conspecific during the Forced Social or Object interaction test (*n* = 6–7/group). Boxplots represent median, upper and lower quartiles with 10th and 90th percentile whiskers. Mean is represented with a cross. ^∗^*p* < 0.05, ^∗∗^*p* < 0.01, ^∗∗∗^*p* < 0.001.

We investigated the role of mGlu5 receptors in sociability using the classical three-chambered social task apparatus, where sociability is measured as the preference for interacting with an enclosed conspecific placed in one of the side chambers as compared to a novel object (an empty cage) placed in the opposite side chamber. At first, we examined germ-line *Grm5*-/- mice and compared them to age-matched WT C57BL/6j mice. During the sociability test, both *Grm5*-/- and WT control mice displayed sociability, spending significantly more time in the social chamber than in the novel object chamber [2-way ANOVA: chamber *F*(2,90) = 133, *p* < 0.001; chamber × genotype *F*(2,90) = 8.06, *p* < 0.001; novel object vs. novel mouse chamber: WT: *p* < 0.001; *Grm5*-/-: *p* < 0.001] (Figure 1A,B). However, *Grm5*-/- mice spent less time than WT mice investigating the novel object chamber (*p* < 0.05) and spent significantly more time in the middle chamber during the test (*p* < 0.05). Similarly, time in close proximity to the novel mouse was higher than for the novel object for both genotypes [2-way ANOVA: close interaction *F*(1,60) = 49.7 *p* < 0.001, and genotype *F*(1,60) = 6.97, *p* < 0.05] (Figure 1C), whereas the overall time in close interaction with the conspecific or object did not differ between genotypes [2-way ANOVA: close interaction × genotype *F*(1,60) = 2.52 *p* = 0.11]. *Grm5*-/- mice showed a higher social preference index for both conspecific chamber time [*t*-test: *t*(1,30) = 2.18, *p* < 0.05] (Figure 1D) and time in close interaction with the conspecific [*t*-test: *t*(1,30) = 3.178, *p* < 0.01] (Figure 1E) when compared to control mice. During the test we observed that the total distance traveled by *Grm5*-/- mice was significantly lower than the control animals [*t*-test: *t*(1,30) = 2.32, *p* < 0.05] (Figure 1F). Since *Grm5*-/- mice are known to display normal locomotion (Chiamulera et al., 2001), this effect could be attributed to a reduced exploratory activity. Unlike WT control, *Grm5*-/- mice explored more actively the novel conspecific during the last 5 min of the test [0–5 vs. 5–10 min: WT, paired *t*-test: *t*(1,15) = 0.25, *p* = 0.79; *Grm5*-/-: *t*(1,15) = 2,68, *p* < 0.05] (Figure 1G,H). *Grm5*-/- mice also exhibited a longer latency to explore the side chambers of the apparatus at the beginning of the test [*t*-test: *t*(1,30) = 4.39, *p* < 0.001] (Figure 1I). These findings suggest that gene-targeted deletion of *Grm5* leads to enhanced social interaction, as measured by the higher social preference index, but also to a reduced exploratory activity or enhanced anxiety as suggested by the reduced distance traveled and high latency to explore the side chambers. To assess whether the lower time spent in the object chamber was due to the putative anxiogenic phenotype or an exploration deficit, we performed an additional experiment in which mice were presented only with the novel object (i.e., the empty enclosure), while the opposite chamber of the apparatus was left empty. In the first 5 min of a 30 min session, *Grm5*-/- indeed explored significantly less the object chamber than WT mice [2-way repeated measures ANOVA: time *F*(5,85) = 0.36; genotype *F*(1,17) = 3.66; time × genotype *F*(5,85) = 7.38 *p* < 0.001; 5 min: WT vs. *Grm5*-/-: *p* < 0.001] (Figure 2A). Conversely, in the remaining time of the session the two genotypes showed no difference in time spent in the object chamber (Figure 2A), therefore, showing no generalized deficit in exploration. These findings strongly suggest that *Grm5*-*/*- mice have an axiogenic phenotype.

Thus, we specifically tested these mice for measures of anxiety-like behavior using the light-dark-box test. *Grm5*-/- mice spent indeed significantly less time in the bright side of the box as compared to WT mice [*t*-test: *t*(1,22) = 2.42, *p* < 0.05] (Figure 2B).

Taken together, these results indicate that *Grm5*-/- mice show an anxiogenic phenotype although with a paradoxically enhanced sociability as measured with social ratios.

### Pharmacological Blockade of mGlu5 Receptors Is Anxiolytic Without Affecting Sociability

**FIGURE 3:**
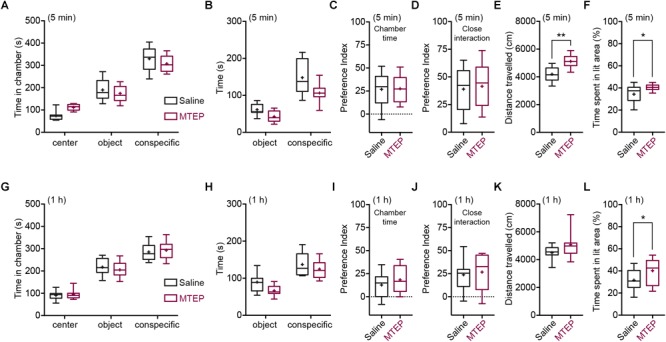
Pharmacological blockade of mGlu5 receptors is anxiolytic without affecting sociability. Time spent (s) in the different compartments of the three-chambered apparatus by MTEP- and vehicle-treated (4% DMSO in saline) mice during the test, at 5 min (MTEP *n* = 13; vehicle *n* = 13) **(A)** or 1 h post-injection (MTEP *n* = 12; vehicle *n* = 12) **(G)**. Time spent (s) in close proximity with the conspecific and object at 5 min **(B)** or 1 h post-injection **(H)**. Preference index derived from the numerical difference between time spent in conspecific and object chamber divided by total time spent × 100 at 5 min **(C)** or 1 h post-injection **(I)**. Index derived from the numerical difference between time spent in close proximity to conspecific and object divided by total time spent in close proximity × 100 at 5 min **(D)** or 1 h post-injection **(J)**. Both social preference indexes did not differ between MTEP and vehicle-treated mice at any post-injection time. At 5 min **(E)** post-injection, but not at 1 h **(K)**, MTEP-treated mice traveled a longer distance than vehicle-treated mice. In the light-dark test, both at 5 min (MTEP *n* = 13; vehicle *n* = 13) **(F)** and 1 h (MTEP *n* = 14; vehicle *n* = 14) **(L)** post-injection, mice treated with MTEP displayed anxiolytic-like activity, spending more time (%) in the lit area of the box than vehicle-treated mice. Boxplots represent median, upper and lower quartiles with 10th and 90th percentile whiskers. Mean is represented with a cross. ^∗^*p* < 0.05, ^∗∗^*p* < 0.01.

We next assessed the effects of mGlu5 receptor negative allosteric modulation in WT C57BL/6J mice in sociability and anxiety. We assessed the effects of MTEP (10 mg/kg) at two different time points, when MTEP receptor occupancy should be at its peak (5–15 min post-i.p. injection) and when it should have returned at baseline levels (1 h post-i.p. injection) (Anderson et al., 2003). Vehicle- and MTEP-treated animals displayed sociability, spending more time in the conspecific chamber than in the novel object chamber both at 5 min [2-way ANOVA: chamber *F*(2,72) = 233, *p* < 0.001; treatment *F*(1,72) = 0.002, *p* > 0.05; and chamber × treatment *F*(2,72) = 4.58, *p* < 0.05; object chamber vs. conspecific chamber: WT *p* < 0.001, *Grm5*-/-*p* < 0.001] and 1 h post-injection [2-way ANOVA: chamber *F*(2,66) = 196.8, *p* < 0.001; treatment *F*(1,66) = 0.001, *p* > 0.05; chamber × treatment *F*(2,66) = 0.59, *p* > 0.05] (Figure 3A,G). MTEP had no effects on the overall time spent in the novel conspecific or novel object chamber either at 5 min (WT vs. *Grm5*-/-: *p* > 0.05) or at 1 h post-injection (WT vs. *Grm5*-/-: *p* > 0.05) (Figure 3A,G). However, MTEP reduced the amount of time spent interacting closely with the conspecific and object in a non-specific manner at 5 min [2-way ANOVA: treatment *F*(1,48) = 13.6, *p* < 0.05; close interaction *F*(1,48) = 84.9, *p* < 0.05; treatment × close interaction *F*(1,48) = 1,93, *p* = 0.17] (Figure 3B) and at 1 h post-injection [2-way ANOVA: treatment *F*(1,44) = 6.14, *p* < 0.05; close interaction *F*(1,44) = 54.8, *p* < 0.05; treatment × close interaction *F*(1,44) = 0.53, *p* = 0.47] (Figure 3H). Social preference indexes for both conspecific chamber time and time in close interaction with the conspecific did not differ between vehicle and MTEP-treated animals both at 5 min [chamber: *t*-test: *t*(1,24) = 0.102, *p* > 0.05; close interaction: *t*-test: *t*(1,24) = 0.312, *p* > 0.05] (Figure 3C,D) and 1 h post-injection [chamber: *t*-test: *t*(1,22) = 0.95, *p* > 0.05; close interaction: *t*-test: *t*(1,22) = 0.36, *p* > 0.05] (Figure 3I,J). These findings indicate that the interaction with a conspecific is not altered by acute MTEP treatment. The lowered active exploration of the conspecific in mice treated with MTEP was accompanied by a marked decrease in object exploration and an increase in locomotor activity during the test, as shown by the distance traveled during the test at 5 min post-injection [*t*-test: *t*(1,24) = 4.56, *p* < 0.001] (Figure 3E) as well as by a statistical trend toward significance at 1 h post injection [*t*-test: *t*(1,22) = 1.77, *p* = 0.09] (Figure 3K).

We then tested whether the reduced active exploration of the object and conspecific during the test was due to the proposed anxiolytic activity of MTEP (Klodzinska et al., 2004; Stachowicz et al., 2007; Lee et al., 2017, 2018; but see Ade et al., 2016). Similar to sociability, we tested the effects of MTEP at both 5 and 1 h post-injection in the light-dark test. MTEP-treated animals spent significantly more time in the bright side of the box, both at 5 min [*t*(1,24) = 2.55, *p* < 0.05] (Figure 3F) and at 1 h post-injection [*t*(1,26) = 2.08, *p* < 0.05] (Figure 3L). Taken together, these results confirm the anxiolytic action of MTEP and suggest that mGlu5 receptor NAM does not influence sociability in WT mice when administered acutely.

### Aberrant Brain Region-Specific Activation After Social Exposure in *Grm5*-/- Mice

**FIGURE 4:**
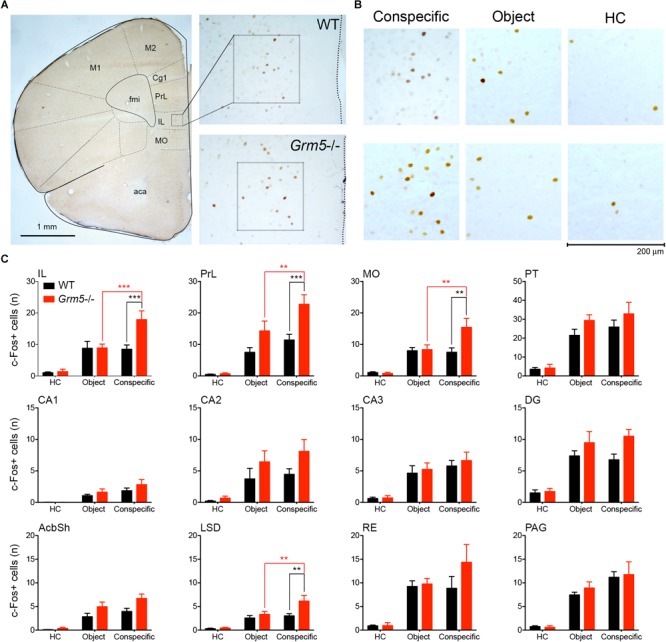
Aberrant brain region-specific activation after social exposure in *Grm5*–/– mice. Number of c-Fos+ cells in WT and *Grm5*–/– mice following 10 min home cage exposure to a conspecific enclosed in a wire mesh cage (conspecific group), a wire mesh cage only (object group) or left undisturbed in home cage (HC, home cage group). **(A)** The micrograph on the left side shows a coronal section immunostained for c-Fos obtained from the frontal lobe of a WT mouse exposed to a novel conspecific in its home cage. A 200 μm × 200 μm sampling window placed in the IL is shown. On the right side, the micrographs show a magnified view of the IL area from where the sampling window was taken in WT (upper panel) and *Grm5*–/– mice (lower panel). **(B)** Representative images of c-Fos immunostaining in the IL in each experimental condition. Scale bar, 200 μm. **(C)** Histograms of the number of c-Fos+ cells for each experimental group in 12 selected brain regions. *N* = 7 in each experimental group. Bars represent mean ± SEM. ^∗^*p* < 0.05, ^∗∗^*p* < 0.01, ^∗∗∗^*p* < 0.001, following two-way ANOVA with Neuman–Keuls *post hoc* comparisons whenever interaction group × genotype resulted significant (see Table 1). Aca, anterior commissure anterior part; AcbSh, accumbens nucleus shell; CA1-3, dorsal hippocampal areas; Cg1, cingulate cortex area 1; DG, dentate gyrus; fmi, forceps minor of the corpus callosum; IL, infralimbic cortex; LSD, lateral septal nucleus dorsal part; M1, primary motor cortex; M2, secondary motor cortex; MO, medial orbital cortex; PAG, periaqueductal gray; PrL, prelimbic cortex; PT, paratenial thalamic nucleus; RE, reuniens thalamic nucleus.

In order to investigate whether the increased sociability observed in Grm5-/- mice could result from different patterns of brain activation upon exposure to social and non-social cues, we exposed WT and *Grm5*-/- mice in their home cage to either a novel conspecific or novel object and quantified the expression of the immediate early gene c-Fos. We used three independent experimental groups for each genotype: a group exposed to an age-matched conspecific enclosed into a wire mesh cage (conspecific group), a group exposed only to the wire mesh cage (object group), and a third control group left undisturbed in the home cage (HC). Similar to the three-chambered social task, Grm5-/- mice displayed a reduced exploration of the novel object in comparison to WT [2-way ANOVA: cue *F*(1,22) = 6.05; genotype *F*(1,22) = 2.45; cue × genotype *F*(1,22) = 4.80, *p* < 0.05; WT vs. *Grm5*-/- object: *p* < 0.05] and a similar exploration of the novel conspecific (Figure 2C). Two hours after the exposure, mice were perfused and processed for immunocytochemistry. Twenty-one brain regions, previously reported to be activated after social interaction (Kim et al., 2015), were preselected for c-Fos analysis. In mice kept undisturbed in their home cage, no significant differences between WT and *Grm5*-/- mice were detected in the number of c-Fos+ neurons in any of the areas analyzed (see Table 1 for statistical significance, Figure 4 and Supplementary Figure S1). This suggests that under resting conditions basal activity in the set of brain areas that we have analyzed is not altered by the lack of mGlu5 receptors. Conversely, compared to the HC group a significant increase in the number of c-Fos+ cells was observed in most of the areas analyzed with the exception of AcbC, LSI, CA1, CA2, and PMCo in WT mice after exposure to a novel object; AcbC, LSI, CA2, and PMCo in WT mice after exposure to a conspecific and AcbC, LSI, CA1, and PMCo in *Grm5*-/-mice after exposure to a novel object (see Table 1).

**Table 1:** Statistical summary data of Figure 3.

		MO	PrL	IL	AcbC	AcbSh	LSD	LSI	MS	MPOM	Pir	PT	RE	LH	BLA	PV	CA1	CA2	CA3	DG	PMCo	PAG
Interaction *F*(2,36)	*F*	4.6	3,9	5,1	1,7	1,7	3,4	1,8	0,7	3,2	0,8	0,6	1,1	0,04	0,9	1,5	0,6	0,7	0,08	1,5	0,3	0,2
	*p*	^∗^	^∗^	^∗^	ns	ns	^∗^	ns	ns	ns	ns	ns	ns	ns	ns	ns	ns	ns	ns	ns	ns	ns
Group *F*(2,36)	*F*	26	34,4	26,5	13,4	28,8	24,4	11,8	42,6	40,4	31,3	30,2	16,1	40,7	22,8	51,6	14,1	11,2	19,7	30,7	20,1	34,1
	*p*	^∗∗∗^	^∗∗∗^	^∗∗∗^	^∗∗∗^	^∗∗∗^	^∗∗∗^	^∗∗∗^	^∗∗∗^	^∗∗∗^	^∗∗∗^	^∗∗∗^	^∗∗∗^	^∗∗∗^	^∗∗∗^	^∗∗∗^	^∗∗∗^	^∗∗∗^	^∗∗∗^	^∗∗∗^	^∗∗∗^	^∗∗∗^
Genotype *F*(1,36)	*F*	4.6	13,9	5,8	3,5	9,1	7,2	3,4	1,3	15,0	0,6	3,2	1,5	0,3	0,5	4,3	1,9	4,6	0,5	5,9	0,9	0,3
	*p*	^∗^	^∗∗∗^	^∗∗^	ns	^∗∗^	^∗^	ns	ns	ns	ns	ns	ns	ns	ns	^∗^	ns	^∗^	ns	^∗^	ns	ns
HC WT vs. HC *Grm5*-/-	*p*	ns	ns	ns	ns	ns	ns	ns	ns	ns	ns	ns	ns	ns	ns	ns	ns	ns	ns	ns	ns	ns
HC WT vs. Obj WT	*p*	^∗∗^	^∗^	^∗^	ns	^∗^	^∗^	ns	^∗∗∗^	^∗∗^	^∗∗^	^∗∗^	^∗^	^∗∗∗^	^∗^	^∗∗∗^	ns	ns	^∗^	^∗∗^	ns	^∗∗^
HC WT vs. Consp WT	*p*	^∗∗^	^∗∗^	^∗∗^	ns	^∗∗^	^∗∗∗^	ns	^∗∗∗^	^∗∗∗^	^∗∗∗^	^∗∗∗^	^∗^	^∗∗∗^	^∗∗∗^	^∗∗∗^	^∗^	ns	^∗^	^∗∗^	^∗∗^	^∗∗∗^
HC *Grm5*-/- vs. Obj *Grm5*-/-	*p*	^∗∗^	^∗∗^	^∗^	ns	^∗∗∗^	^∗∗^	ns	^∗∗∗^	^∗∗^	^∗∗^	^∗^	^∗^	^∗∗∗^	^∗∗^	^∗∗∗^	ns	^∗^	^∗∗^	^∗∗∗^	ns	^∗∗∗^
HC *Grm5*-/- vs. Consp *Grm5*-/-	*p*	^∗∗∗^	^∗∗^	^∗∗∗^	^∗∗∗^	^∗∗∗^	^∗∗∗^	^∗∗∗^	^∗∗^	^∗∗∗^	^∗∗∗^	^∗∗∗^	^∗∗∗^	^∗∗∗^	^∗∗∗^	^∗∗∗^	^∗∗∗^	^∗∗^	^∗∗∗^	^∗∗∗^	^∗∗∗^	^∗∗∗^
Obj WT vs. Consp WT	*p*	ns	ns	ns	ns	ns	ns	ns	ns	ns	ns	ns	ns	ns	ns	ns	ns	ns	ns	ns	ns	ns
Obj *Grm5*-/- vs. Consp *Grm5*-/-	*p*	^∗∗^	^∗∗^	^∗∗∗^	^∗^	Ns	^∗∗^	^∗^	^∗∗^	ns	ns	ns	ns	ns	ns	ns	ns	ns	ns	ns	^∗^	ns
Obj WT vs. Obj *Grm5*-/-	*p*	ns	ns	ns	ns	ns	ns	ns	ns	^∗∗^	ns	ns	ns	ns	ns	ns	ns	ns	ns	ns	ns	ns
Consp WT vs. Consp *Grm5*-/-	*p*	^∗∗^	^∗∗∗^	^∗∗∗^	^∗^	^∗^	^∗∗^	^∗^	ns	^∗∗^	ns	ns	ns	ns	ns	ns	ns	ns	ns	ns	ns	ns

*^∗^p < 0.05, ^∗∗^p < 0.01, ^∗∗∗^p < 0.001. Two-way ANOVAs followed by Newman–Keuls comparisons.*

A significant group × genotype interaction in the 2-way ANOVAs was observed only in 4 brain areas, namely IL, MO, PrL, and LSD (see Table 1 and Figure 4). The interaction with a conspecific triggered higher activation as compared with the object in *Grm5*-/- mice, whereas no statistical significant differences were observed between object and conspecific exposed-groups in WT mice. These four brain areas had also a higher number of c-Fos+ cells in *Grm5*-/- mice as compared to WT mice when exposed to the novel conspecific, but not to the novel object (see Table 1 and Figure 4). The arousal produced by the exposure to the novel object or conspecific in our paradigm may have masked in WT mice distinct pattern of c-Fos activation. On the other hand, the lack of mGlu5 receptors was able to induce region-specific changes in the number of c-Fos+ cells specifically related to social interaction. We, thus, reasoned that social interaction, contrary to non-social, rather than producing a higher degree of activation, namely number of c-Fos+ cells per area, it enhances functional connectivity among a set of brain regions. In addition, the high activation of prefrontal areas and LSD observed in *Grm5*-/- mice, could underlie a disrupted activity coordination. To explore this possibility, we analyzed the functional connectivity generated during social or object investigation in WT and *Grm5*-/- mice.

### Altered Functional Connectivity of Multiple Brain Regions During Social Exposure in *Grm5*-/- Mice

**FIGURE 5:**
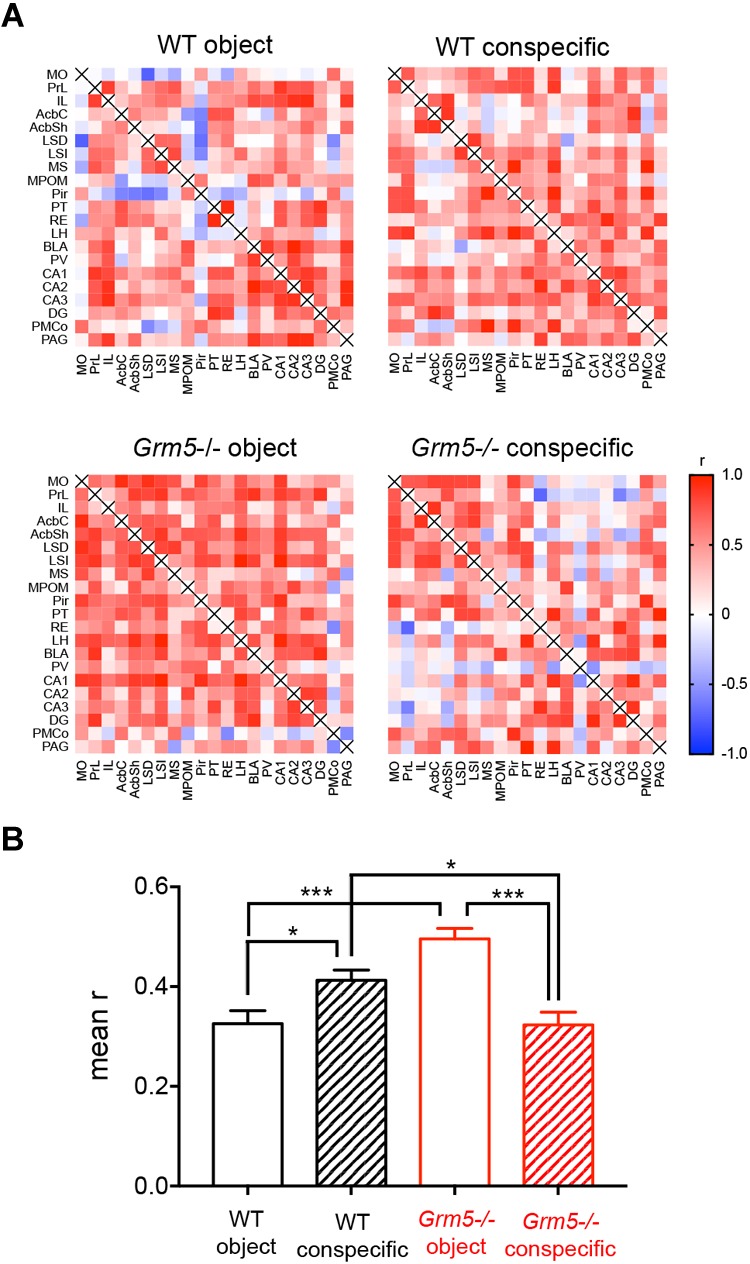
Altered functional connectivity of multiple brain regions during social exposure in *Grm5*–/– mice. **(A)** Matrices displaying interregional correlations of c-Fos expression in WT and *Grm5*–/– mice exposed to an object (wire mesh cage) or to an enclosed conspecific. Colors represent the correlation strength based on Pearson’s *r* scale. Warmer colors show stronger correlations. **(B)** Mean *r* calculated from each genotype and experimental condition matrix. Bars represent mean ± SEM. ^∗^*p* < 0.05, ^∗∗^*p* < 0.01, ^∗∗∗^*p* < 0.001.

In order to infer interactions between neural elements, we computed correlation coefficients across subjects using our c-Fos expression data set (Horwitz et al., 1995; Tanimizu et al., 2017a,b; Rogers-Carter et al., 2018). This allowed us to obtain an approximation of the strength of the coordinated activity changes among brain regions following social and non-social interactions in *Grm5*-/- and WT mice. We first computed inter-regional correlations for each experimental group (Figure 5A). As shown in Figure 5B, changes in network density upon social investigation were observed both in WT and *Grm5*-/- mice [2-way ANOVA: genotype *F*(1,836) = 6.50, *p* < 0.05; group *F*(1,836) = 6.66, *p* < 0.01; group × genotype *F*(1,836) = 47.51, *p* < 0.001]. A higher functional connectivity (mean *r*) was observed upon conspecific as compared to object interaction in WT mice (*p* < 0.05). Conversely, in *Grm5*-/- mice functional connectivity was higher upon non-social interaction (*p* < 0.001). Interaction with a conspecific led to higher functional connectivity in WT mice as compared to *Grm5*-/- mice (*p* < 0.05).

These results suggest that in WT mice social investigation leads to a higher functional connectivity than upon interaction with an object. On the contrary, the lack of mGlu5 receptors inverts the strength of the functional connectivity toward non-social interaction.

### Social Interaction Network Hubs Are Rearranged in *Grm5*-/- Mice

**FIGURE 6:**
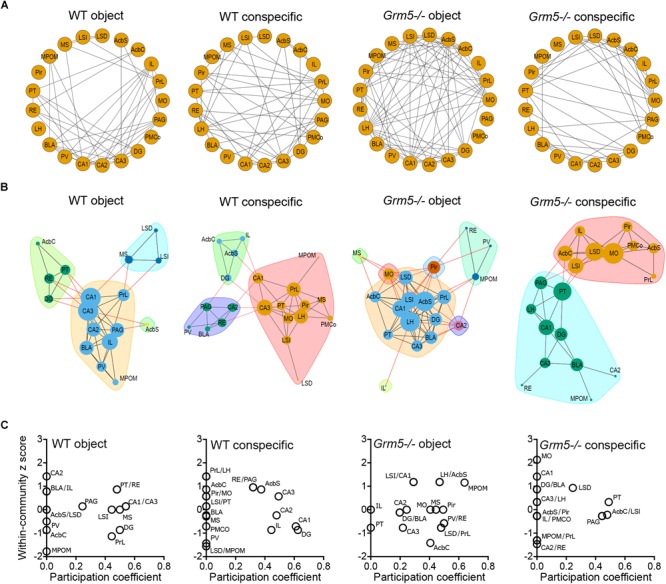
Social interaction network hubs are rearranged in *Grm5*–/– mice. **(A)** Network graphs showing above-threshold (*r* > 0.6) significant positive correlations (*p* < 0.05) between the different brain regions in each experimental condition and genotype. **(B)** Networks identified for each experimental condition and genotype. The width of the edges is proportional to the correlation strength of above-threshold significant correlations and the node size is proportional to the degree (number of edges associated with a given node) of the node in a given network. Colors in the network represent the different communities identified via modularity optimization. **(C)** Within-community *z* scores and participation coefficients of each brain region in the different networks generated following interaction with an object or conspecific in WT and *Grm5*–/– mice.

Based on interregional matrices (Figure 5A), we generated network graphs (Figure 6A), where nodes represent brain regions and edges represent above-threshold (Pearson’s *r* > 0.6) significant (*p* < 0.05) correlations (Figure 6A). We further applied graph theoretical analysis to the network graphs to explore if social investigation induces changes to the relative weight of any of the identified nodes and whether they are affected by the lack of mGlu5 receptors (Figure 6B). We then computed the within-community *z* score and participation coefficient for each node (Figure 6C). In correlation-based networks, the within-community *z* score measures how well connected a region is to its own community and the participation coefficient of a node constitutes a measure of the degree to which a node is linked with nodes in other communities (Guimerà and Amaral, 2005). The participation coefficient, therefore, denotes “hubness” (Power et al., 2013; Rogers-Carter et al., 2018). Nodes displaying a high value of within-community *z* score and participation coefficient are thought to be key hubs, crucial for coordinating other nodes and, thus, the overall activity of the network (Tanimizu et al., 2017a). Our analysis identified the RE, AcbSh, PAG, and CA3 as key hubs in the social interaction functional network and the PT in the object functional network in WT mice. In addition, the hippocampal regions CA1, CA2, and DG as well as the IL appear as key regions in coordinating the activity between communities in the social interaction network given their high participation coefficient. Of note is the differential participation of the PrL and IL in the object and conspecific functional networks, respectively. In *Grm5*-/- mice, we observed a complete derangement in the role of nodes in both the non-social and social functional networks. In these animals, while the hippocampus and mPFC lost their coordinating role in conspecific network activity, the lateral septum increased it. Moreover, the PT transferred its role as a key hub from the non-social to the social functional network.

## Discussion

Here, we reassessed how genetic ablation and pharmacological blockade of mGlu5 receptors affect sociability and anxiety-like behavior in mice. We show that germ line deletion of mGlu5 receptors leads to an anxiogenic phenotype accompanied by a paradoxical enhancement of sociability. Conversely, negative allosteric modulation of this receptor reduced anxiety-like behavior in the light-dark test, consistent with previous studies (Klodzinska et al., 2004; Stachowicz et al., 2007; Lee et al., 2017, 2018), without influencing sociability in WT mice. We further determined how the lack of mGlu5 receptors affects the pattern of brain activation following social and non-social interaction by quantifying the number of neurons expressing the IEG c-Fos. A computational approach was then used to model the potential impact of mGlu5 receptor ablation on functional connectivity of brain areas relevant for social interaction. Our c-Fos quantification revealed a restricted activation, limited to the MO, PrL, IL, and LSD, in *Grm5*-/- mice following interaction with a conspecific as compared to a novel object, although the anxiogenic phenotype of these animals could have in part influenced our analysis. On the other hand, the lack of wide-ranging changes in cFos expression between social and non-social interactions in control mice might have been confounded by the high arousal state induced by the alteration of the home cage environment. Alternatively, our computational analysis suggests that social interaction, rather than inducing broad changes in c-Fos expression, leads to an increased functional connectivity of specific brain regions important for social behavior.

As a first step toward understanding the role of mGlu5 receptors in social preference, we examined the behavior of *Grm5*-/- mice in the three-chambered social task. We observed that ablation of the mGlu5 receptor enhances sociability, as indicated by the social preference index. This was accompanied by an increase in anxiety-like behavior, that was initially observed as a delayed exploration of the side chambers and a reduced distance traveled in the three-chambered social task. This was then confirmed by the light-dark test in which *Grm5*-/- mice spent less time in the lit compartment than WT mice. The anxiogenic phenotype observed in *Grm5*-/- mice appears at odds with the widely accepted anxiolytic action of mGlu5 receptor antagonists (see for review: Ferraguti, 2018) and the reduced stress-induced hyperthermia previously reported for these mice (Brodkin et al., 2002). *Grm5*-/- mice were previously shown to explore more the center of a novel arena in the open field test, but were found to behave similarly to control animals in the light-dark and elevated plus-maze tests (Olsen et al., 2010). However, Inta et al. (2013) reported an age-dependent anxiogenic phenotype in *Grm5*-/- mice using the light-dark test, consistent with our findings. These controversies could be explained by differences in the anxiety tests used, e.g., conflict-based vs. physiological measurement, or by procedural variations. Since *Grm5*-/- mice have a normal locomotor activity (Chiamulera et al., 2001), we can exclude that the reduced time spent in the lit compartment of the light-dark test could have resulted from a motor impairment.

Inhibition of mGlu5 receptor activity through systemic administration of antagonists was found to rescue the impaired social behavior typical of the BTBR inbred mouse strain and of different mouse models of ASD (Silverman et al., 2012; Gantois et al., 2013; Chung et al., 2015). On the other hand, inconsistent effects of mGlu5 antagonists were reported in WT rodents, e.g., MTEP induced social isolation in rats (Koros et al., 2007), whereas MPEP increased sniffing and extended time spent interacting in Balb/c mice, but reduced sniffing in Swiss Webster mice (Burket et al., 2011).

In our study, the three-chambered social task revealed increased social preference in *Grm5*-/- mice, based on the preference index, despite the total time spent in the social chamber was similar to that of WT animals. However, given the increased anxiety-like behavior showed by *Grm5*-/- mice, the sociability expressed by these animals in the three-chambered social task might have been underestimated. Selective ablation of the mGlu5 receptor in parvalbumin-positive neurons resulted in higher duration of social interaction bouts (Barnes et al., 2015), whereas their ablation in cortical glutamatergic principal cells did not produce any detectable effect (Jew et al., 2013). The increased sociability in germline *Grm5*-/- mice may, therefore, primarily result from a role of mGlu5 receptors at inhibitory circuits. Further studies should explore the pathways and neurons at which mGlu5 receptors regulate sociability.

Our pharmacological study shows that negative allosteric modulation of mGlu5 receptors with MTEP had no effect on sociability despite it reduced active exploration of both the conspecific and object. These non-specific effects of MTEP on social and non-social interactions could explain the reported social isolation in rats after MTEP treatment (Koros et al., 2007). The complex effects on measures of sociability observed in mice (Burket et al., 2011), on the other hand, may depend on the known off-target effects of MPEP (Lea and Faden, 2006). The anxiolytic action elicited by MTEP in WT mice in our study, although modest, confirms that the absence of an effect on sociability does not result from a lack of activity of the drug.

Both mGlu5 receptor NAMs MPEP and MTEP have been described as potent anxiolytics in different rodent models (see for review: Ferraguti, 2018). However, their anxiolytic properties on WT mice appear to greatly depend on strain, dose and delay between administration and testing. MTEP was found to be anxiolytic at 3 mg/kg and anxiogenic at 30 mg/kg in the light-dark test in C57BL/6j (Lee et al., 2018). Whether this differential effect on anxiety is due to an inverted U-shaped dose-response activity or to potential unspecific effects of the highest dose of MTEP remains to be explored.

From these findings, it could be concluded that deviations in any direction of mGlu5 receptor function may lead to impairments in both social and anxiety-like behaviors.

To understand at the anatomical level where mGlu5 receptors regulate brain activity during social exploration, we have analyzed the expression of c-Fos in a selected set of brain areas previously reported to be activated upon social interaction (Kim et al., 2015). Our study shows that in *Grm5*-/- mice, MO, PrL, IL, and LSD were selectively activated upon interaction with a conspecific, suggesting that mGlu5 receptors dampen neuronal activity in these brain regions during social behavior, possibly by activating interneurons and facilitating feedforward inhibition (Pollard et al., 2014). Future studies will have to unveil the expression and role of mGlu5 receptors in the different neuronal types in these brain areas and their specific contribution to social behavior.

Our functional connectivity analysis of 21 brain regions relevant for social behavior suggested a disruption of the network density in *Grm5*-/- during exploration of both social and non-social stimuli. Moreover, the networks generated during social and non-social interactions, as well as the role of individual brain regions in coordinating network activity, such as hippocampal and prefrontal areas, dramatically changed in *Grm5*-/- mice. In line with previous findings investigating prolonged social interactions (Tanimizu et al., 2017a), we observed high participation coefficients of the hippocampus and mPFC in WT mice exposed to a conspecific. It should be taken into account, however, that c-Fos correlation-based networks suffer from several limitations. Inclusion of different brain regions into the network can lead to rearrangements of the communities, measures of centrality and roles of individual nodes. Thus, with current computational models it is hard to compare the role of single nodes between differently generated networks or even between similar experiments that include different brain regions into the network. Nonetheless, c-Fos based functional connectivity and network analysis can serve as a promising tool for hypothesis generation (Vetere et al., 2017; Rogers-Carter et al., 2018), although promising key hubs will have to be validated experimentally, e.g., using chemo- or opto-genetic approaches.

The hippocampus and mPFC, together with the amygdala, appear as key regions underlying sociability circuits (Felix-Ortiz and Tye, 2014; Felix-Ortiz et al., 2016). The CA2 region, in particular, has been recently proposed as a critical hub for socio-cognitive memory processing (Hitti and Siegelbaum, 2014), independent of other hippocampus-dependent behaviors such as spatial memory. In our study, the CA2 region was shown to display largely different roles in the functional networks generated following a 10 min interaction with a conspecific or an object, sufficient time to allow for social and non-social memory formation (Tanimizu et al., 2017a,b). In the network generated upon interaction with a conspecific in *Grm5*-/- mice, the CA2 region was the most isolated node in the network. Therefore, it would be of interest to determine whether manipulation of mGlu5 receptors activity in this region affects social recognition memory as predicted by our functional network analysis.

To our knowledge, only one study has attempted to address the contribution of mGlu5 receptors in a specific brain region to sociability, so far. In line with our findings that the lack of mGlu5 receptors increases activity in the LSD upon social interaction, Mesic et al. (2015) suggested that selective removal of mGlu5 receptors in this brain area impaired expression of sociability but not social novelty.

In conclusion, our work shows that while the mGlu5 receptor NAM MTEP is anxiolytic upon systemic acute administration, the lack of these receptors, as in germ line *Grm5*-/- mice, results in an anxiogenic phenotype. Similarly, while sociability is not affected by pharmacological blockade of mGlu5 receptors, their lack leads to an apparent increased sociability. Whether this is the result of complex and perhaps opposite effects at different brain regions or developmental adaptations remains to be established. Indeed, a further note of caution concerns data obtained with germ line knockouts as adaptive changes may influence behavior differently from manipulations carried out in adult animals. Further studies in which mGlu5 receptor activity is abolished or modulated in a time-controlled and region- and/or cell-type specific manner are, therefore, warranted. Our c-Fos expression and network analyses offers candidate areas for such specific targeting.

Taken together our findings support the relevance of mGlu5 receptors in modulating anxiety-like behavior and sociability. The paradoxical increased social preference within an overall anxiogenic phenotype, as in the germ line Grm5-/- mice, shows remarkable analogies with Williams syndrome (Meyer-Lindenberg et al., 2006; Barak and Feng, 2016). Future studies should address whether mGlu5 function is altered in this rare neurodevelopmental disorder extending the implication of these receptors beyond ASD.

## Author Contributions

AR-P and FF conceived and designed the project. AR-P, JK, MZ, EP, and GS were involved with experimental and analytical aspects of the manuscript. AR-P performed data analyses, functional connectivity, and graph theoretical analysis. AR-P and FF wrote the manuscript. All contributing authors commented on the manuscript.

## Conflict of Interest Statement

The authors declare that the research was conducted in the absence of any commercial or financial relationships that could be construed as a potential conflict of interest.

## Supplementary Material

FIGURE S1 Histograms of the number of c-Fos+ cells for each experimental group in the other 9 selected brain regions. *N* = 7 in each experimental group. Bars represent mean ± SEM. ^∗^*p* < 0.05, ^∗∗^*p* < 0.01, ^∗∗∗^*p* < 0.001, following two-way ANOVA with Neuman–Keuls *post hoc* comparisons whenever interaction group × genotype resulted significant (see Table 1). AcbC, accumbens nucleus core; BLA, basolateral amygdala; LH, lateral hypothalamic area; LSI, lateral septal nucleus intermedial part; MPOM, medial preoptic nucleus medial part; MS, medial septal nucleus; Pir, piriform cortex; PMCo, posteromedial cortical amygdaloid area; PV, paraventricular thalamic nucleus.
