# Supplementary figures and images for: An Appraisal of the Influence of the Metabotropic Glutamate 5 (mGlu5) Receptor on Sociability and Anxiety
Source: Front Mol Neurosci. 2019 Feb 19;12:30. doi: 10.3389/fnmol.2019.00030 (PMC6401637; doi:10.3389/fnmol.2019.00030)

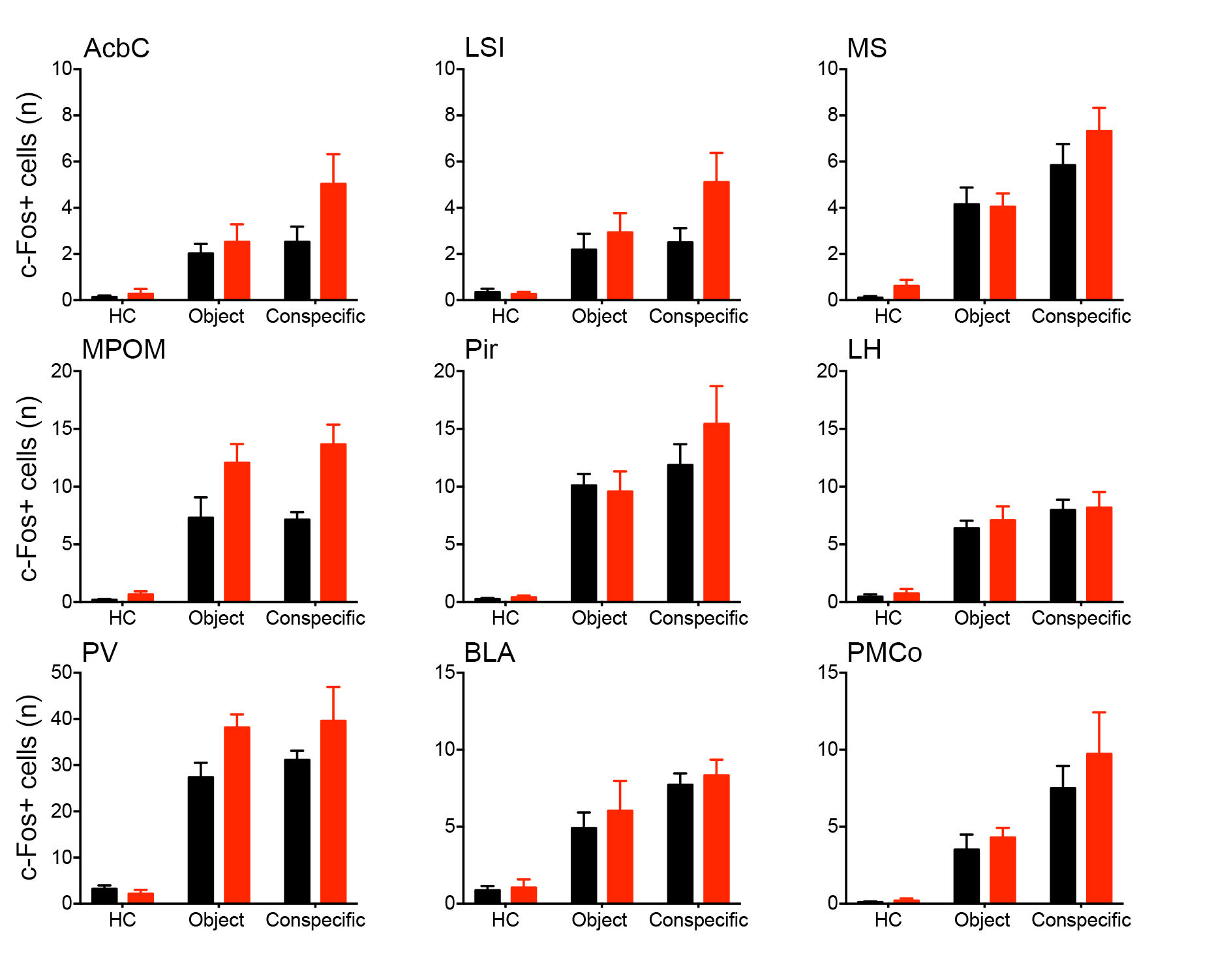

Supplement: FIGURE S1 — Histograms of the number of c-Fos+ cells for each experimental group in the other 9 selected brain regions. N = 7 in each experimental group. Bars represent mean ± SEM. ∗p < 0.05, ∗∗p < 0.01, ∗∗∗p < 0.001, following two-way ANOVA with Neuman–Keuls post hoc comparisons whenever interaction group × genotype resulted significant (see Table 1). AcbC, accumbens nucleus core; BLA, basolateral amygdala; LH, lateral hypothalamic area; LSI, lateral septal nucleus intermedial part; MPOM, medial preoptic nucleus medial part; MS, medial septal nucleus; Pir, piriform cortex; PMCo, posteromedial cortical amygdaloid area; PV, paraventricular thalamic nucleus. [file Image_1.TIF]
